# The associations between affective states and motivation in Tunisian cross country trained runners

**DOI:** 10.3389/fpsyg.2026.1877487

**Published:** 2026-07-10

**Authors:** Najha Bouallagui, Chiraz Goumni, Said Ben Hsan, Raouf Hammami, Riadh Khalifa, Imen Ben Amar

**Affiliations:** 1 University of Manouba, Higher Institute of Sport and Physical Education of Ksar-Said, University Campus, Manouba, Tunisia; 2 Research Laboratory (LR23JS01) “Sport Performance, Health & Society”, Tunis, Tunisia; 3 Tunisian Research Laboratory “Sports Performance Optimization”, National Center of Medicine and Science in Sports (CNMSS-LR09SEP01), Tunis, Tunisia

**Keywords:** affective states, behavior, motivation, psychological states, self-determination theory

## Abstract

**Introduction:**

This study draws on Self-Determination Theory to examine the relationships between motivational regulations and pre-competition affective states in Tunisian adolescent competitive cross-country runners, with consideration of gender differences.

**Methods:**

A total of 116 athletes participated in the study (56% female; 44% male; *M* = 16.31 years). Participants completed the French versions of the Sport Motivation Scale-II (SMS-II/EMS-II) and the Positive and Negative Affect Schedule (PANAS-D).

**Results:**

Correlation analyses revealed significant associations between motivational regulations and affective states, with coefficients ranging from *r* = 0.30 to *r* = 0.86. In general, autonomous forms of motivation were positively associated with Positive Affect and negatively associated with Negative Affect, whereas controlled forms of motivation showed the opposite pattern. Multiple regression analyses indicated that the model for Positive Affect was statistically significant, *F*(6, 109) = 92.139, *p* < 0.001, *R*^2^ = 0.835. Intrinsic motivation (*B* = 0.544, *SE* = 0.175, *β* = 0.308, 95% CI [0.197, 0.891], *p* = 0.002), integrated regulation (*B* = 0.514, *SE* = 0.228, *β* = 0.218, 95% CI [0.063, 0.966], *p* = 0.026), and external regulation (*B* = −0.630, *SE* = 0.234, β = −0.290, 95% CI [−1.094, −0.167], *p* = 0.008) emerged as significant predictors. The model for Negative Affect was also significant, *F*(6, 109) = 53.856, *p* < 0.001, *R*^2^ = 0.748, with only external regulation as a significant predictor (*B* = 1.380, *SE* = 0.272, *β* = 0.677, 95% CI [0.842, 1.919], *p* < 0.001).

**Conclusion:**

These findings indicate that motivational regulations are collectively associated with pre-competition affective states, yet only specific forms of regulation make independent contributions when examined simultaneously. The results support Self-Determination Theory by highlighting the differentiated roles of autonomous and controlled motivation in relation to athletes’ emotional experiences in a Tunisian endurance sport context.

## Introduction

The investigation of psychological determinants of athletic performance has become a major focus in contemporary sport science. In endurance disciplines such as cross-country running, athletes are continuously exposed to intense physiological and psychological demands requiring sustained effort, tolerance to fatigue, emotional control, and persistence under pressure. Consequently, psychological factors play a decisive role in athletes’ engagement, performance, and long-term well-being. Among these factors, affective states and motivational regulations have emerged as two key constructs influencing athletic functioning and adaptation (Hardy et al., 2017; Hanin, 2000).

Affective states refer to the emotional experiences and mood-related responses individuals encounter in everyday life and sport settings. In competitive sport, athletes may experience a broad spectrum of emotions ranging from positive feelings such as enthusiasm, confidence, excitement, and enjoyment to negative emotions including anxiety, tension, frustration, and fatigue. The Positive and Negative Affect Schedule (PANAS) developed by Watson et al. (1988) is widely used to assess these emotional dimensions. These affective experiences are not merely transient reactions; rather, they influence cognitive functioning, attentional processes, physiological activation, and behavioral responses (Fredrickson, 2001). In endurance sports specifically, the ability to maintain positive affective states while effectively regulating negative emotions has been associated with enhanced performance, greater resilience, and reduced risk of psychological exhaustion or burnout (Campo et al., 2017).

In parallel, motivation represents another central psychological determinant of sport participation and performance. Self-Determination Theory (SDT) proposed by Deci and Ryan (1985) provides one of the most influential frameworks for understanding human motivation in sport contexts. According to SDT, motivation exists along a continuum ranging from highly self-determined forms to controlled and non-self-determined forms. This continuum includes intrinsic motivation, integrated regulation, identified regulation, introjected regulation, external regulation, and amotivation. Intrinsic motivation reflects engagement in sport for inherent pleasure and satisfaction, whereas integrated and identified regulations involve participation based on personal values and self-endorsement. In contrast, introjected and external regulations are driven by internal pressures or external rewards and constraints, while amotivation reflects a lack of intentionality and purpose toward participation (Vallerand, 2007a,b). Previous research has consistently demonstrated that autonomous forms of motivation are associated with greater enjoyment, persistence, psychological well-being, and superior long-term outcomes in athletes (Ryan and Deci, 2017).

From a Self-Determination Theory perspective, motivational regulations are theorized to influence athletes’ emotional experiences by shaping how they perceive, interpret, and respond to sport participation. Athletes characterized by autonomous forms of motivation are more likely to experience enjoyment, satisfaction, and feelings of competence, which promote positive affective states. In contrast, controlled forms of motivation and amotivation have been associated with greater psychological distress, tension, and negative emotional experiences (Ryan and Deci, 2017; Vallerand, 2007a,b). Consequently, motivation may be viewed as an important antecedent of affective states, providing a theoretical basis for examining the extent to which different motivational regulations predict positive and negative affect among athletes. This perspective is particularly relevant in endurance sports, where athletes are regularly confronted with prolonged physical discomfort and psychological challenges during training and competition.

Despite increasing scientific interest in these psychological constructs, relatively few studies have examined the extent to which motivational regulations are associated with affective states in endurance athletes, particularly within non-Western and underrepresented cultural contexts. Tunisian adolescent competitive cross-country runners constitute an especially relevant population for investigation due to the unique sociocultural, environmental, and sporting characteristics influencing athletic development and competitive experiences. Furthermore, sport psychology research conducted in North African contexts remains limited compared with European and North American settings.

Therefore, the present study aimed to investigate the relationships between motivational regulations and affective states among Tunisian adolescent competitive cross-country runners. More specifically, guided by Self-Determination Theory, the study examined whether different forms of motivational regulation predict positive and negative affect. By exploring these associations within a specific cultural and sporting context, this study seeks to contribute to the existing literature and provide practical implications for coaches, sport psychologists, and practitioners seeking to develop culturally sensitive interventions aimed at enhancing athletes’ emotional well-being, motivation, and competitive performance.

## Methods

### Study design

This study adopted a cross-sectional correlational design grounded in Self-Determination Theory (SDT) to examine the relationships between pre-competition affective states and different forms of sport motivation among competitive adolescent athletes. The study also explored whether these relationships varied according to gender. A total of 116 Tunisian competitive adolescent endurance athletes participated in the study, including 56% females and 44% males, with a mean age of 16.31 years. Participants were recruited from endurance sport disciplines through sports clubs and training centres. Eligibility criteria required athletes to be actively engaged in competitive sport and regularly participating in official competitions.

Data were collected during the pre-competition phase under standardised conditions. Participants completed the French versions of the Sport Motivation Scale-II (SMS-II/EMS-II) and the Positive and Negative Affect Schedule (PANAS-D). The SMS-II assesses six motivational regulations derived from SDT, namely intrinsic motivation, integrated regulation, identified regulation, introjected regulation, external regulation, and amotivation, using a 7-point Likert scale ranging from 1 (“does not correspond at all”) to 7 (“corresponds exactly”). The PANAS-D was used to evaluate positive affect and negative affect experienced before competition. Both instruments have demonstrated satisfactory psychometric properties in French-speaking sport contexts.

Participants completed the questionnaires individually in the presence of the researchers or trained assistants. Pearson correlation analyses were performed to examine the relationships between motivational regulations and affective states. Multiple linear regression analyses were subsequently conducted to assess the predictive contribution of motivational regulations to positive affect and negative affect.

### Participants

A total of 116 Tunisian cadet cross-country runners participated in the present study. Participants were aged between 16 and 17 years (M = 16.3; SD = 0.465), including 65 females (56%) and 51 males (44%). All athletes regularly represented their clubs in national competitions and had an average training experience of 3.16 years (SD = 1.387). Participants were recruited from endurance sport disciplines through sports clubs and training centres. Eligibility criteria required athletes to be actively engaged in competitive sport and regularly participating in official competitions. A non-probability quota sampling method was used.

Prior to participation, all athletes were informed about the aims of the study, the voluntary nature of participation, and the confidentiality of their responses. Written informed consent was obtained from all participants, and from parents or legal guardians in the case of minors, in accordance with ethical standards for research involving human participants. The study was conducted in accordance with the latest version of the Declaration of Helsinki, and the protocol was approved by the Local Ethics Committee of the National Centre of Medicine and Science of Sports, Tunis (CNMSS-LR09SEP01) prior to commencement. None of the participating athletes reported a history of psychological, musculoskeletal, neurological, or orthopaedic disorders within 6 months prior to data collection.

### Procedures

Data collection was conducted through collaboration with the Tunisian Athletics Federation during the 2021–2022 competitive season. Following authorization from the technical director, statistical information regarding registered athletes was obtained, and appointments were organized with club presidents and coaches. Athletes were then invited to voluntarily complete the questionnaires after providing their informed consent. To ensure consistency in emotional assessment, participants completed the questionnaires within the 2 hours preceding their competition.

### Demographic information

Participants were asked to provide demographic information including age, gender, and the number of years of sport practice.

### Positive and negative affect schedule including a direction scale (PANAS-D)

Affective states were assessed using the French version of the Positive and Negative Affect Schedule including a Direction Scale (PANAS-D; Nicolas et al., 2014). The original PANAS was developed by Watson et al. (1988) to provide a simple, reliable, and economical instrument for assessing affective states, consistent with the bidimensional structure of affect proposed in previous theoretical and empirical works (Diener et al., 1985; Russell, 1980, 1983; Watson et al., 1984; Watson and Tellegen, 1985; Zevon and Tellegen, 1982).

Over time, several alternative versions of the PANAS have been developed, including the Positive and Negative Affect Schedule-Expanded Form (PANAS-X; Watson and Clark, 1994), which contains 60 adjectives, the Positive and Negative Affect Schedule Short Form (PANAS-SF; Kercher, 1992), and the International Positive and Negative Affect Schedule Short Form (I-PANAS-SF; Thompson, 2007). Despite these alternatives, the PANAS remains one of the most widely used instruments for assessing affective states in sport psychology research.

The PANAS-D was used to assess athletes’ affective states before competition. The scale consists of four subscales evaluating: Positive Affect intensity (10 items), Negative Affect intensity (10 items), Positive Affect direction (10 items), and Negative Affect direction (10 items). Athletes were asked to rate: (a) the intensity of each affective symptom on a 5-point Likert scale ranging from 1 (“not at all” or “very slightly”) to 5 (“extremely”); and (b) the degree to which each affective state was perceived as facilitative or debilitative for subsequent performance using a 7-point Likert scale ranging from −3 (“very debilitative”) to +3 (“very facilitative”).

### Sport motivation scale-II (SMS-II / EMS-II)

Sport motivation was assessed using the French version of the Sport Motivation Scale-II (SMS-II/EMS-II), originally developed and validated by Pelletier et al. (2013), and subsequently examined by Li et al. (2018), Stenling et al. (2015), and Nascimento Junior et al. (2014). The French adaptation was provided by Pelletier et al. (2019).

Grounded in Self-Determination Theory (SDT; Deci and Ryan, 1985, 2000), the SMS-II assesses six forms of motivational regulation in sport: intrinsic motivation, integrated regulation, identified regulation, introjected regulation, external regulation, and amotivation. The questionnaire contains 18 items rated on a 7-point Likert scale ranging from 1 (“do not agree at all”) to 7 (“completely agree”). Participants were asked to indicate the extent to which each statement corresponded to their reasons for practicing sport.

The SMS-II is currently considered one of the most widely used and validated instruments for assessing athletes’ motivation across various sport settings (Clancy et al., 2017).

### Statistical analyses

Data analyses were performed using IBM SPSS Statistics version 25 (IBM Corp, 2017). Descriptive statistics, including means (M), standard deviations (SD), minimum and maximum values, skewness, and kurtosis, were calculated for all study variables. The normality of data distribution was assessed using skewness and kurtosis indices according to the recommendations of Field (2013) and Tabachnick and Fidell (2013). In addition, the Kolmogorov–Smirnov test was used to verify normality assumptions.

The internal consistency of the PANAS-D and EMS-II subscales was evaluated using Cronbach’s alpha coefficients. Values ≥ 0.70 were considered indicative of acceptable reliability (Nunnally, 1978).

Independent-samples *t*-tests were conducted to examine gender differences in affective states and motivational regulations. In addition to *p*-values, Cohen’s d effect sizes were calculated to assess the magnitude of between-group differences and interpreted according to conventional thresholds: trivial (<0.20), small (0.20–0.49), medium (0.50–0.79), and large (≥0.80) (Cohen, 1988).

Pearson’s product–moment correlation coefficients (r) were computed to examine the relationships between affective states and motivational regulations (Tabachnick and Fidell, 2013).

Finally, multiple linear regression analyses were performed to determine the extent to which motivational regulations predicted affective states. Prior to conducting the regression analyses, multicollinearity assumptions were evaluated using tolerance values and Variance Inflation Factors (VIF), which indicated no severe violations (Tolerance > 0.20; VIF < 5) (Kutner et al., 2005). Tolerance values greater than 0.10 and VIF values below 10 were considered indicative of acceptable levels of multicollinearity (Field, 2013). Standardized regression coefficients (*β*), coefficients of determination (R^2^), and adjusted R^2^ values were examined to evaluate the relative contribution of each predictor and the overall explanatory power of the models. Statistical significance was set at *p* < 0.05 for all analyses.

## Results

### Descriptive statistics and reliability

Table 1 presents the descriptive statistics, scale ranges, distribution characteristics, and internal consistency coefficients for all study variables. Regarding affective states assessed by the PANAS-D, athletes reported relatively high levels of Positive Affect (M = 30.82 ± 10.26), whereas lower scores were observed for Negative Affect (M = 21.43 ± 10.98), including NA anxiety (M = 11.23 ± 4.83) and NA hostility (M = 10.09 ± 5.07). Concerning motivational regulations assessed by the EMS-II, participants generally reported higher scores for autonomous forms of motivation, particularly more self-determined regulation (M = 25.07 ± 6.94), compared with controlled forms of motivation and amotivation.

**Table 1:** Descriptive statistics, distribution characteristics, and reliability coefficients for PANAS-D and EMS-II subscales.

Variable	Range	Min	Max	Skewness	Kurtosis	M	SD	α
Positive affect	10–50	11	46	−0.489	−1.280	30.82	10.26	0.923
Negative affect	10–50	10	50	0.855	−0.708	21.43	10.98	0.938
NA anxiety	5–25	5	24	0.835	−0.392	11.23	4.83	0.866
NA hostility	5–25	5	25	0.956	−0.190	10.09	5.07	0.897
Intrinsic motivation	3–21	3	21	−0.344	−1.392	13.86	5.80	0.935
Integrated regulation	3–21	5	20	−0.243	−1.295	12.98	4.34	0.912
Identified regulation	3–21	5	20	0.318	−0.277	12.09	3.12	0.759
More self-determined regulation	6–42	12	38	−0.161	−1.017	25.07	6.94	0.897
Introjected regulation	3–21	5	21	0.723	−0.362	11.05	4.19	0.709
External regulation	3–21	4	21	0.759	−0.910	9.17	4.72	0.882
Less self-determined regulation	6–42	10	42	0.789	−0.803	21.09	9.54	0.899
Amotivation	3–21	3	14	0.408	−0.737	7.59	3.05	0.807

Cronbach’s alpha coefficients ranged from 0.86 to 0.94 for the PANAS-D subscales and from 0.71 to 0.94 for the EMS-II subscales, indicating satisfactory to excellent internal consistency for all measures.

### Gender differences

Independent-samples *t*-tests were conducted to examine gender differences in affective states and motivational regulations (Table 2). Significant differences were observed for identified regulation (*t* = −3.289, *p* = 0.001), more self-determined regulation (*t* = −2.235, *p* = 0.027), and amotivation (*t* = −2.257, *p* = 0.026), with male athletes reporting higher scores than female athletes in these variables. No statistically significant gender differences were found for Positive Affect, Negative Affect, intrinsic motivation, integrated regulation, introjected regulation, external regulation, or less self-determined regulation (all *p* > 0.05), indicating overall similarity between male and female athletes for most study variables.

**Table 2:** Gender differences in PANAS-D and EMS-II subscales with effect sizes (Cohen’s d).

Subscales	Female (*n* = 65) M ± SD	Male (*n* = 51) M ± SD	*t*	*p*	Cohen’s *d*
Positive affect	30.78 ± 9.89	30.86 ± 10.80	−0.041	0.968	0.01
Negative affect	21.06 ± 10.72	21.90 ± 11.39	−0.408	0.684	0.08
NA anxiety	10.97 ± 4.60	11.57 ± 5.14	−0.662	0.509	0.12
NA hostility	10.05 ± 4.84	10.14 ± 5.39	−0.096	0.924	0.02
Intrinsic motivation	13.29 ± 5.56	14.59 ± 6.07	−1.196	0.234	0.22
Integrated regulation	12.54 ± 4.16	13.55 ± 4.53	−1.249	0.214	0.23
Identified regulation	11.28 ± 2.45	13.12 ± 3.57	−3.289	0.001*	0.62
More self-determined regulation	23.82 ± 6.16	26.67 ± 7.58	−2.235	0.027*	0.42
Introjected regulation	11.12 ± 4.02	10.96 ± 4.43	0.206	0.837	−0.04
External regulation	9.05 ± 4.62	9.33 ± 4.89	−0.324	0.746	0.06
Less self-determined regulation	21.09 ± 9.44	21.10 ± 9.77	−0.003	0.997	0.00
Amotivation	7.03 ± 2.87	8.29 ± 3.15	−2.257	0.026*	0.42

To complement statistical significance testing, effect sizes (Cohen’s d) were calculated and reported in Table 2 to provide information on the magnitude of observed differences.

### Correlation analysis

Pearson correlation analyses revealed significant associations between motivational regulations and affective states (Table 3). Autonomous forms of motivation, including intrinsic motivation, integrated regulation, and identified regulation, were positively associated with Positive Affect (*r* = 0.692–0.869, *p* < 0.01) and negatively associated with Negative Affect dimensions (*r* = −0.619 to −0.810, *p* < 0.01).

**Table 3:** Pearson correlation coefficients between PANAS-D and EMS-II subscales.

Variables	1	2	3	4	5	6	7	8	9	10	11	12
1Positive affect	—											
2Negative affect	−0.836**	—										
3NA anxiety	−0.753**	0.945**	—									
4NA hostility	−0.802**	0.965**	0.890**	—								
5Intrinsic motivation	0.869**	−0.810**	−0.761**	−0.773**	—							
6Integrated regulation	0.852**	−0.796**	−0.765**	−0.739**	0.901**	—						
7Identified regulation	0.692**	−0.666**	−0.619**	−0.666**	0.714**	0.726**	—					
8More self-determined reg.	0.844**	−0.797**	−0.756**	−0.761**	0.884**	0.951**	0.903**	—				
9Introjected regulation	−0.782**	0.724**	0.669**	0.712**	−0.722**	−0.702**	−0.609**	−0.712**	—			
10External regulation	−0.868**	0.864**	0.818**	0.833**	−0.839**	−0.819**	−0.701**	−0.827**	0.854**	—		
11Less self-determined reg.	−0.858**	0.838**	0.786**	0.809**	−0.820**	−0.799**	−0.681**	−0.805**	0.958**	0.954**	—	
12Amotivation	−0.396**	0.377**	0.421**	0.308**	−0.362**	−0.402**	−0.212*	−0.346**	0.304**	0.481**	0.387**	—

In contrast, controlled forms of motivation, including introjected and external regulation, as well as amotivation, showed negative associations with Positive Affect (*r* = −0.396 to −0.868, *p* < 0.01) and positive associations with Negative Affect dimensions (*r* = 0.308–0.864, *p* < 0.01). Overall, these results indicate that more self-determined motivational profiles are associated with more adaptive affective states.

### Multiple regression analysis

Multiple linear regression analyses were conducted to examine the extent to which motivational regulations were associated with affective states (Table 4). Prior to analysis, multicollinearity diagnostics were assessed using tolerance and Variance Inflation Factor (VIF) values. Tolerance values ranged from 0.130 to 0.682, and VIF values ranged from 1.466 to 7.673, all remaining within acceptable thresholds (VIF < 10; tolerance > 0.10), indicating no serious multicollinearity concerns (Kutner et al., 2005).

**Table 4:** Multiple regression analyses predicting affective states from motivational regulations.

Predictor	B	SE	*β*	*t*	*p*	95% CI	Tolerance	VIF
Positive affect								
Intrinsic motivation	0.544	0.175	0.308	3.109	0.002*	[0.197, 0.891]	0.154	6.494
Integrated regulation	0.514	0.228	0.218	2.260	0.026*	[0.063, 0.966]	0.163	6.128
Identified regulation	0.073	0.199	0.022	0.369	0.713	[−0.321, 0.467]	0.414	2.416
Introjected regulation	−0.348	0.188	−0.142	−1.849	0.067	[−0.721, 0.025]	0.256	3.910
External regulation	−0.630	0.234	−0.290	−2.694	0.008*	[−1.094, −0.167]	0.130	7.673
Amotivation	−0.033	0.159	−0.010	−0.209	0.835	[−0.347, 0.281]	0.682	1.466
*R^2^* = 0.835; Adjusted *R^2^* = 0.826; *F*(6, 109) = 92.139, *p* < 0.001								
Negative affect								
Intrinsic motivation	−0.245	0.203	−0.148	−1.207	0.230	[−0.648, 0.157]	0.154	6.494
Integrated regulation	−0.264	0.264	−0.119	−1.000	0.320	[−0.788, 0.260]	0.163	6.128
Identified regulation	−0.153	0.231	−0.049	−0.662	0.509	[−0.610, 0.305]	0.414	2.416
Introjected regulation	−0.172	0.219	−0.075	−0.789	0.432	[−0.606, 0.261]	0.256	3.910
External regulation	1.380	0.272	0.677	5.079	<0.001*	[0.842, 1.919]	0.130	7.673
Amotivation	−0.130	0.184	−0.041	−0.704	0.483	[−0.494, 0.235]	0.682	1.466
*R^2^* = 0.748; Adjusted *R^2^* = 0.734; *F*(6, 109) = 53.856, *p* < 0.001								

*N* = 116. B, unstandardized regression coefficient; SE, standard error; β, standardized regression coefficient; 95% CI, 95% confidence interval for B; VIF, Variance Inflation Factor. Standard errors and 95% confidence intervals are reported to facilitate interpretation of the precision of regression estimates. Tolerance and VIF diagnostics are reported to evaluate the extent of multicollinearity among motivational regulation predictors. VIF values ranged from 1.466 to 7.673 (all < 10), and tolerance values ranged from 0.130 to 0.682 (all > 0.10), indicating that multicollinearity was within acceptable limits and did not invalidate the regression results (Kutner et al., 2005). *p* values < 0.05 are marked with an asterisk.

The regression model predicting Positive Affect was statistically significant, *F*(6, 109) = 92.139, *p* < 0.001, and explained 83.5% of the variance (*R*^2^ = 0.835, Adjusted *R*^2^ = 0.826). Within this model, intrinsic motivation (*B* = 0.544, *SE* = 0.175, *β* = 0.308, 95% CI [0.197, 0.891], *p* = 0.002), integrated regulation (*B* = 0.514, *SE* = 0.228, *β* = 0.218, 95% CI [0.063, 0.966], *p* = 0.026), and external regulation (*B* = −0.630, *SE* = 0.234, *β* = −0.290, 95% CI [−1.094, −0.167], *p* = 0.008) emerged as significant predictors. Identified regulation (*B* = 0.073, *SE* = 0.199, *β* = 0.022, 95% CI [−0.321, 0.467], *p* = 0.713), introjected regulation (*B* = −0.348, *SE* = 0.188, *β* = −0.142, 95% CI [−0.721, 0.025], *p* = 0.067), and amotivation (*B* = −0.033, *SE* = 0.159, *β* = −0.010, 95% CI [−0.347, 0.281], *p* = 0.835) were not statistically significant predictors.

Similarly, the regression model predicting Negative Affect was statistically significant, *F*(6, 109) = 53.856, *p* < 0.001, explaining 74.8% of the variance (*R*^2^ = 0.748, Adjusted *R*^2^ = 0.734). However, only external regulation significantly predicted Negative Affect (*B* = 1.380, *SE* = 0.272, *β* = 0.677, 95% CI [0.842, 1.919], *p* < 0.001). Intrinsic motivation (*B* = −0.245, *SE* = 0.203, *β* = −0.148, 95% CI [−0.648, 0.157], *p* = 0.230), integrated regulation (*B* = −0.264, *SE* = 0.264, *β* = −0.119, 95% CI [−0.788, 0.260], *p* = 0.320), identified regulation (*B* = −0.153, *SE* = 0.231, *β* = −0.049, 95% CI [−0.610, 0.305], *p* = 0.509), introjected regulation (*B* = −0.172, *SE* = 0.219, *β* = −0.075, 95% CI [−0.606, 0.261], *p* = 0.432), and amotivation (*B* = −0.130, *SE* = 0.184, *β* = −0.041, 95% CI [−0.494, 0.235], *p* = 0.483) were not significant predictors in the multivariate model.

## Discussion

The present study examined the relationships between motivational regulations and pre-competition affective states in Tunisian adolescent competitive cross-country runners, with a specific focus on the predictive role of Self-Determination Theory (SDT) motivational constructs. The results revealed substantial associations between motivational regulations and affective states, with correlation coefficients ranging from *r* = 0.30 to *r* = 0.86. In general, autonomous forms of motivation were positively associated with Positive Affect and negatively associated with Negative Affect, whereas controlled forms of motivation and amotivation showed the opposite pattern. These findings are consistent with SDT assumptions (Deci and Ryan, 1985; Ryan and Deci, 2017) and prior sport psychology research highlighting the emotional benefits of self-determined motivation (Vallerand, 2007a,b; Ntoumanis, 2012).

However, the regression analyses provided a more nuanced understanding of these relationships. Although motivational regulations collectively explained a substantial proportion of variance in affective states (82% for Positive Affect and 76% for Negative Affect), only specific motivational regulations emerged as significant independent predictors when controlling for shared variance among variables. Specifically, intrinsic motivation, integrated regulation, and external regulation significantly predicted Positive Affect, whereas only external regulation significantly predicted Negative Affect. Other forms of regulation, including identified regulation, introjected regulation, and amotivation, did not make significant unique contributions in the regression models. These findings highlight the importance of distinguishing between bivariate associations and multivariate predictive effects when interpreting motivation–affect relationships.

The very high explained variance observed in both models should also be interpreted with caution. Such magnitudes may partly reflect conceptual overlap among SDT motivational regulations, as these constructs are theoretically adjacent and empirically interrelated. In addition, the exclusive reliance on self-report questionnaires for both predictors and outcomes may have inflated associations due to shared method variance. Common source bias and response tendencies may therefore have contributed to the strength of the observed predictive relationships (Podsakoff et al., 2003; Richardson et al., 2009). These methodological considerations should be taken into account when interpreting the robustness of the regression estimates.

The significant positive role of intrinsic motivation and integrated regulation in predicting Positive Affect supports SDT propositions that self-determined motivation is associated with enjoyment, vitality, and positive emotional functioning. Athletes who engage in sport for inherent pleasure or because it is congruent with their personal identity are more likely to experience positive emotional states before competition. These findings are consistent with previous research demonstrating that autonomous forms of motivation are associated with greater well-being, positive emotions, persistence, and adaptive psychological functioning in sport contexts (Ntoumanis, 2012; Ryan and Deci, 2020). In contrast, the finding that external regulation significantly predicts both Positive Affect (negatively) and Negative Affect (positively) underscores the emotional cost of controlled motivational processes, where participation is driven by external pressures, rewards, or obligations. Controlled forms of motivation have previously been associated with psychological need frustration, emotional distress, and maladaptive outcomes such as athlete burnout (Bartholomew et al., 2011; Lonsdale et al., 2009).

The absence of significant unique effects for identified regulation, introjected regulation, and amotivation in the regression models suggests that their influence on affective states may be largely shared with other motivational dimensions. This indicates that, in this sample of adolescent endurance athletes, the motivational–affective relationship is better understood as a multivariate system rather than isolated linear effects of each regulation type. It also reinforces the importance of considering multicollinearity and shared variance when interpreting SDT-based motivational profiles.

From a cultural and contextual perspective, the present findings contribute to a more nuanced understanding of motivation in North African sport settings. Tunisian adolescent endurance athletes operate within a sporting environment shaped by strong social expectations, family involvement, and coach-centered training structures, which may emphasize external outcomes such as performance ranking, selection, and recognition. Such contextual pressures may partly explain the relatively strong role of external regulation in predicting affective states, particularly negative emotional experiences. At the same time, the presence of meaningful associations involving intrinsic motivation and integrated regulation suggests that self-determined forms of motivation remain relevant and influential even within more controlling or performance-oriented environments.

Moreover, adolescence represents a critical developmental stage for these athletes, where identity formation and social approval are particularly salient. In the Tunisian context, success in sport may carry strong symbolic value for social mobility and recognition, potentially intensifying both autonomous and controlled motivational processes. This cultural framing may help explain the high explained variance observed in the regression models, as motivational dynamics in this population are likely embedded within dense social and performance-related expectations.

These findings extend previous SDT research by providing evidence from an underrepresented cultural context. While SDT is considered a universal theory, growing evidence suggests that the expression, internalization, and behavioral consequences of motivational regulations are shaped by sociocultural conditions and socialization processes (Chirkov et al., 2003; Ryan and Deci, 2020). The present results therefore highlight both the universality of the motivation–affect relationship and its contextual specificity within Tunisian endurance sport.

From a practical perspective, the results underline the importance of fostering autonomous motivational climates in endurance sport settings. Coaches and practitioners should prioritize strategies that enhance athletes’ sense of autonomy, competence, and relatedness, while reducing excessive reliance on external pressures. Promoting intrinsic enjoyment and internalization of goals may help optimize athletes’ emotional readiness before competition and support long-term engagement.

Several limitations should be acknowledged. The cross-sectional design prevents causal inference between motivational regulations and affective states. In addition, reliance on self-report measures may introduce response bias and shared method variance. Finally, the sample was limited to Tunisian adolescent competitive cross-country runners, which may restrict generalisability to other sports, age groups, or cultural contexts. Future research should consider longitudinal and cross-cultural designs, as well as incorporate additional psychological and contextual variables such as coaching style, perceived motivational climate, and coping strategies.

## Conclusion

In conclusion, this study demonstrates that motivational regulations are strongly associated with pre-competition affective states among Tunisian adolescent competitive cross-country runners. However, only specific motivational regulations independently predict emotional experiences when modeled simultaneously. These findings refine our understanding of Self-Determination Theory in sport by highlighting the differentiated roles of motivational regulations and underscore the importance of cultural and contextual factors in shaping athletes’ psychological functioning.

The present study highlights meaningful relationships between motivational regulations and affective states in Tunisian adolescent competitive cross-country runners. Correlation analyses showed significant associations between PANAS-D and EMS-II subscales, with coefficients ranging from *r* = 0.30 to *r* = 0.86, indicating a consistent link between athletes’ motivational profiles and their emotional experiences.

Multiple regression analyses further demonstrated that motivational regulations collectively explain a substantial proportion of variance in affective states, accounting for 82% of Positive Affect and 76% of Negative Affect in this sample of adolescent endurance athletes. However, when examined simultaneously, only specific motivational regulations contributed independently to these outcomes. Intrinsic motivation, integrated regulation, and external regulation significantly predicted Positive Affect, while external regulation was the only significant predictor of Negative Affect.

Overall, these findings suggest that although athletes’ motivational profiles are strongly associated with their emotional states, the independent influence of each motivational regulation varies when shared variance is controlled. The results emphasize the particular importance of fostering autonomous forms of motivation while reducing externally controlled motivational processes to support more adaptive emotional functioning in adolescent endurance athletes.

From an applied perspective, these findings underline the relevance of developing autonomy-supportive coaching environments in Tunisian endurance sport contexts. Future research should further investigate the contextual and psychological mechanisms underlying these relationships, as well as examine their longitudinal impact on athletes’ emotional well-being and performance.

## Data Availability

The raw data supporting the conclusions of this article will be made available by the authors, without undue reservation.

## References

[ref1] Bartholomew K. J. Ntoumanis N. Ryan R. M. Bosch J. A. Thøgersen-Ntoumani C. (2011). Self-determination theory and diminished functioning: the role of interpersonal control and psychological need thwarting. Personal. Soc. Psychol. Bull. 37, 1459–1473. doi: 10.1177/0146167211413125 21700794

[ref2] Campo M. Mellalieu S. D. Martin J. (2017). Coping in sport: a systematic review of the literature. Int. Rev. Sport Exerc. Psychol. 10, 1–52. doi: 10.1080/1750984X.2016.119029927695511

[ref3] Chirkov V. Ryan R. M. Kim Y. Kaplan U. (2003). Differentiating autonomy from individualism and independence: a self-determination theory perspective on internalization of cultural orientations and well-being. J. Pers. Soc. Psychol. 84, 97–110. doi: 10.1037/0022-3514.84.1.97, 12518973

[ref4] Clancy R. B. Herring M. P. Campbell M. J. (2017). Measures of motivation in sport: critical review and bibliometric analysis. Front. Psychol. 8:348. doi: 10.3389/fpsyg.2017.00348 28337165 PMC5343045

[ref9001] Cohen J. (1988). Statistical Power Analysis for the Behavioral Sciences (2nd ed.). Hillsdale, NJ: Lawrence Erlbaum Associates.

[ref5] Deci E. L. Ryan R. M. (1985). Intrinsic Motivation and Self-Determination in human Behavior. New York: Plenum Press.

[ref6] Deci E. L. Ryan R. M. (2000). The “what” and “why” of goal pursuits: human needs and the self-determination of behavior. Psychol. Inq. 11, 227–268. doi: 10.1207/S15327965PLI1104_01

[ref7] Diener E. Larsen R. J. Levine S. Emmons R. A. (1985). Intensity and frequency: dimensions underlying positive and negative affect. J. Pers. Soc. Psychol. 48, 1253–1265. doi: 10.1037/0022-3514.48.5.1253, 3998989

[ref8] Field A. (2013). Discovering Statistics using IBM SPSS Statistics. 4th Edn London: SAGE Publications.

[ref9] Fredrickson B. L. (2001). The role of positive emotions in positive psychology: the broaden-and-build theory of positive emotions. Am. Psychol. 56, 218–226. doi: 10.1037/0003-066X.56.3.218 11315248 PMC3122271

[ref10] Hanin Y. L. (2000). Emotions in Sport. Champaign: Human Kinetics.

[ref11] Hardy L. Jones G. Gould D. (2017). Understanding Psychological Preparation for Sport: Theory and Practice. Chichester: John Wiley & Sons.

[ref12] IBM Corp (2017). IBM SPSS Statistics for Windows, Version 25.0. Armonk: IBM Corp.

[ref13] Kercher K. (1992). Assessing subjective well-being in the old-old: the PANAS as a measure of orthogonal dimensions of positive and negative affect. Res. Aging 14, 131–168. doi: 10.1177/0164027592142001

[ref14] Kutner M. H. Nachtsheim C. J. Neter J. Li W. (2005). Applied Linear Statistical Models. 5th Edn New York: McGraw-Hill/Irwin.

[ref15] Li C. Kawabata M. Zhang L. (2018). Validity and reliability of the sport motivation scale-II for Chinese athletes. Int. J. Sport Exerc. Psychol. 16, 51–64. doi: 10.1080/1612197X.2016.1153130

[ref16] Lonsdale C. Hodge K. Rose E. A. (2009). Athlete burnout in elite sport: a self-determination perspective. J. Sports Sci. 27, 785–795. doi: 10.1080/02640410902929366 19437185

[ref17] Nascimento J. R. A. Vissoci J. R. N. Balbim G. M. Moreira C. R. Pelletier L. G. Vieira L. F. (2014). Adaptação transcultural e análise das propriedades psicométricas da Sport Motivation Scale-II no contexto brasileiro. Rev. Educ. Fís. UEM 25, 441–458. doi: 10.4025/reveducfis.v25i3.24855

[ref18] Nicolas M. Martinent G. Campo M. (2014). Evaluation of the psychometric properties of a modified positive and negative affect schedule including a direction scale (PANAS-D) among French athletes. Psychol. Sport Exerc. 15, 227–237. doi: 10.1016/j.psychsport.2014.01.001

[ref19] Ntoumanis N. (2012). “A self-determination theory perspective on motivation in sport and physical education: current trends and possible future research directions,” in Advances in Motivation in Sport and Exercise, eds. Roberts G. C. Treasure D. C. (Champaign: Human Kinetics), 91–128.

[ref20] Nunnally J. C. (1978). Psychometric Theory. 2nd Edn New York: McGraw-Hill.

[ref21] Pelletier L. G. Rocchi M. A. Guertin C. Hébert C. Sarrazin P. (2019). French adaptation and validation of the sport motivation scale-II. Int. Rev. Sport Exerc. Psychol. 17, 232–249. doi: 10.1080/1750984X.2019.1675761

[ref22] Pelletier L. G. Rocchi M. A. Vallerand R. J. Deci E. L. Ryan R. M. (2013). Validation of the revised sport motivation scale (SMS-II). Psychol. Sport Exerc. 14, 329–341. doi: 10.1016/j.psychsport.2012.12.002

[ref23] Podsakoff P. M. MacKenzie S. B. Lee J. Y. Podsakoff N. P. (2003). Common method biases in behavioral research: a critical review of the literature and recommended remedies. J. Appl. Psychol. 88, 879–903. doi: 10.1037/0021-9010.88.5.879, 14516251

[ref24] Richardson H. A. Simmering M. J. Sturman M. C. (2009). A tale of three perspectives: examining post hoc statistical techniques for detection and correction of common method variance. Organ. Res. Methods 12, 762–800. doi: 10.1177/1094428109332834

[ref25] Russell J. A. (1980). A circumplex model of affect. J. Pers. Soc. Psychol. 39, 1161–1178. doi: 10.1037/h0077714

[ref26] Russell J. A. (1983). Pancultural aspects of the human conceptual organization of emotions. J. Pers. Soc. Psychol. 45, 1281–1288. doi: 10.1037/0022-3514.45.6.1281

[ref27] Ryan R. M. Deci E. L. (2017). Self-Determination Theory: Basic Psychological Needs in Motivation, Development, and Wellness. New York: Guilford Press.

[ref28] Ryan R. M. Deci E. L. (2020). Intrinsic and extrinsic motivation from a self-determination theory perspective: definitions, theory, practices, and future directions. Contemp. Educ. Psychol. 61:101860. doi: 10.1016/j.cedpsych.2020.101860

[ref29] Stenling A. Ivarsson A. Johnson U. Lindwall M. (2015). Bayesian structural equation modeling in sport and exercise psychology. J. Sport Exerc. Psychol. 37, 410–420. doi: 10.1123/jsep.2015-0010, 26442771

[ref30] Tabachnick B. G. Fidell L. S. (2013). Using Multivariate Statistics. 6th Edn Boston: Pearson Education.

[ref31] Thompson E. R. (2007). Development and validation of an internationally reliable short-form of the positive and negative affect schedule (PANAS). J. Cross-Cult. Psychol. 38, 227–242. doi: 10.1177/0022022106297301

[ref32] Vallerand R. J. (2007a). “A hierarchical model of intrinsic and extrinsic motivation in sport and exercise,” in Advances in Motivation in sport and Exercise, eds. Roberts G. C. Treasure D. C. (Champaign: Human Kinetics), 255–279.

[ref33] Vallerand R. J. (2007b). “Intrinsic and extrinsic motivation in sport and physical activity: a review and a look at the future,” in Handbook of Sport Psychology, eds. Tenenbaum G. Eklund R. C. (Hoboken: Wiley), 59–83.

[ref34] Watson D. Clark L. A. (1994). The PANAS-X: Manual for the Positive and Negative Affect Schedule–Expanded Form. Iowa City: University of Iowa.

[ref35] Watson D. Clark L. A. Tellegen A. (1984). Cross-cultural convergence in the structure of mood: a Japanese replication and comparison with American findings. J. Pers. Soc. Psychol. 47, 127–144. doi: 10.1037/0022-3514.47.1.127

[ref36] Watson D. Clark L. A. Tellegen A. (1988). Development and validation of brief measures of positive and negative affect: the PANAS scales. J. Pers. Soc. Psychol. 54, 1063–1070. doi: 10.1037/0022-3514.54.6.1063, 3397865

[ref37] Watson D. Tellegen A. (1985). Toward a consensual structure of mood. Psychol. Bull. 98, 219–235. doi: 10.1037/0033-2909.98.2.219, 3901060

[ref38] Zevon M. A. Tellegen A. (1982). The structure of mood change: an idiographic/nomothetic analysis. J. Pers. Soc. Psychol. 43, 111–122. doi: 10.1037/0022-3514.43.1.111

